# On The Microstructures and Hardness of The Nb-24Ti-18Si-5Al-5Cr-5Ge and Nb-24Ti-18Si-5Al-5Cr-5Ge-5Hf (at.%) Silicide Based Alloys

**DOI:** 10.3390/ma12172655

**Published:** 2019-08-21

**Authors:** Zifu Li, Panos Tsakiropoulos

**Affiliations:** Department of Materials Science and Engineering, The University of Sheffield, Sir Robert Hadfield Building, Mappin Street, Sheffield S1 3JD, UK

**Keywords:** Nb-silicide based alloys, high entropy alloys, complex concentrated alloys, macrosegregation, intermetallics, hardness

## Abstract

The microstructures and hardness of the as cast and heat treated (1400 °C/100 h) alloys Nb-24Ti-18Si-5Ge-5Cr-5Al (ZF6) and Nb-24Ti-18Si-5Ge-5Cr-5Al-5Hf (ZF9) were studied. Both alloys were compared with refractory metal bcc solid solution + intermetallic High Entropy Alloys (HEAs). There was macrosegregation of Si, Ti, Cr and Al in both alloys. The roles of Ge and Hf on macrosegregation are discussed. In both alloys the primary phase was the βNb_5_Si_3_. In the as cast alloy ZF6 the Nb_ss_, βNb_5_Si_3_ and C14-NbCr_2_ Laves phase and Nb_ss_ + βNb_5_Si_3_ eutectic were formed. The microstructure of the as cast alloy ZF9 consisted of Nb_ss_, βNb_5_Si_3_, γNb_5_Si_3_ and C14-NbCr_2_ Laves phase. The heat-treated microstructures of the alloys ZF6 and ZF9 consisted of Nb_ss_, βNb_5_Si_3_ and αNb_5_Si_3_ and Nb_ss_, βNb_5_Si_3,_ αNb_5_Si_3_ and γNb_5_Si_3_, respectively. The surfaces of both alloys were contaminated by oxygen where TiO_2_ and HfO_2_ formed respectively in the alloys ZF6 and ZF9. Alloying with Hf increased the lattice parameter of Nb_ss_ and decreased the hardness of ZF9 and Nb_5_Si_3_. The roles of alloying additions on the hardness of the Nb_ss_ and Nb_5_Si_3_ and relationships between alloy hardness and alloy parameters VEC (valence electron concentration), δ (related to atomic size) and Δχ (related to electronegativity) were discussed.

## 1. Introduction

The search for new alloys capable of operating in the demanding conditions in gas turbine engines at temperatures higher than those experienced by the currently used Ni-based superalloys has concentrated on refractory metal silicide-based alloys, in particular Nb-Si based alloys and Mo-Si based alloys [1,2]. The former also are known as Nb-silicide based alloys or Nb in situ composites and have microstructures consisting of Nb_ss_ and intermetallic(s), such as Nb_5_Si_3_, Nb_3_Si, NbCr_2_ and others, depending on alloy chemistry and processing [2,3]. 

The tetragonal Nb_5_Si_3_ silicide is most desirable compared with Nb_3_Si owing to its better creep properties and oxidation resistance. Alloying additions can enhance the stability of the tetragonal βNb_5_Si_3_, suppress the Nb_ss_ + Nb_3_Si eutectic and replace it by the Nb_ss_ + βNb_5_Si_3_ eutectic, enhance the eutectoid decomposition of Nb_3_Si and modify the properties and morphology of the silicides [3,4].

Important alloying additions in Nb-silicide based alloys are Al, Cr, Ti and Hf. These four elements are essential for oxidation resistance [3,5], the first two are expected to confer oxidation resistance with Si, and Hf scavenges oxygen and forms hafnia. The solid solubilities of Al, Cr and Hf in the Nb_ss_ increase with increasing Ti content and all four elements strengthen the Nb_ss_. Aluminium and Cr increase the ductile to brittle transition temperature (DBTT) of bcc Nb. Aluminium has a stronger effect than Cr, and Ti and Hf have a weak effect on DBTT [6]. Alloying with Cr and Ti respectively increases and decreases the shear modulus of Nb. Aluminium and Cr additions in Nb or Nb-Ti increase the Peierls-Nabarro energy and thus reduce dislocation mobility, tensile ductility and thereby decrease the resistance to fracture of the Nb_ss_ [7]. 

Titanium and Hf substitute Nb in Nb_5_Si_3_ and can destabilise its desirable tetragonal structure [2,3], and decrease its hardness [8]. In Nb_5_Si_3_ the solid solubility of Cr is low, and Si can be substituted by Al [8]. Germanium, like Al, substitutes for Si in Nb_5_Si_3_ [9] and in synergy with Ti and Hf improves the fracture toughness of Nb_5_Si_3_ from about 3 MPa√m to 8–11 MPa√m for 24 at.% Ti in solution [10,11]. 

Our research group has shown that in Nb-24Ti-18Si-5Al-5Cr based alloys, the Al and Cr in synergy with Ti stabilise the Nb_ss_ + βNb_5_Si_3_ eutectic, and Al in synergy with Ti enhances the transformation of βNb_5_Si_3_ to αNb_5_Si_3_ and promotes the precipitation of Nb_ss_ via the transformation βNb_5_Si_3_ → αNb_5_Si_3_ + Nb_ss_ [12]. The latter transformation was enhanced even in the absence of Ti when Al and Ge were in synergy with Si in the alloy Nb-18Si-5Al-5Ge [13]. Cretegny et al. have attributed the increased strength of MASC (Nb-24Ti-16Si-8.2Hf-2Cr-1.9Al) type alloys (compared with Nb-25Ti-8Hf-xSi alloys) to the propensity of Al and Cr additions to favour eutectic growth compared with the simpler quaternary alloys [14]. 

The authors have shown that Ge promotes the Nb_ss_ + βNb_5_Si_3_ eutectic and microstructures similar to those achieved in Nb-silicide based alloys where Ti was in synergy with Al and Cr [13,15]. Considering the reported beneficial effect of Ge on the oxidation of Nb-silicide based alloys [5] and the restrictions imposed on the Al, Cr and Hf contents of Nb-24Ti-18Si-zxAl-yCr-zHf based alloys owing to mechanical property targets, we embarked on the work presented in this paper in order to understand how the synergy of Ge with the alloying elements Al, Cr, Ti and Hf at the concentrations of the “base” alloy Nb-24Ti-18Si-5Al-5Cr affects the stability of βNb_5_Si_3_ and NbCr_2_, and the vol.% of Nb_ss_ and the shape and size of Nb_5_Si_3_. This knowledge is important for optimising room and elevated temperature mechanical properties and oxidation resistance [3]. The effects of the synergy of the above elements on the oxidation behaviour of alloys based on Nb-24Ti-18Si-5Al-5Cr will be the subject of a separate publication. In this paper we report on two alloys with Ge and Hf additions. The results for each alloy are presented separately. The as solidified and heat-treated microstructures are compared with similar alloys without Ge. The nominal compositions of the latter alloys are given in the Appendix A. 

## 2. Experimental

The nominal compositions of the alloys of this study (all compositions are given in at.%) were Nb-24Ti-18Si-5Al-5Cr-5Ge (alloy ZF6) and Nb-24Ti-18Si-5Al-5Cr-5Ge-5Hf (alloy ZF9). The Ge, Si and Ti concentrations were set respectively at 5, 18 and 24 at.%, as in previous research [12,13]. The DBTT of aero engine materials must not exceed −50 °C [3]. The Al composition was kept at 5 at.% because the DBTT of Nb increases above −50 °C at higher Al concentrations [6] and the Cr composition was kept at 5 at.% as in previous work [12,15]. This choice of alloys makes possible their comparison with non-Ge containing alloys regarding stability of microstructure (this work) and oxidation (separate publication).

We used arc melting and high purity elements (Nb 99.99 wt.%, Ti 99.95 wt.%, Si 99.999 wt.%, Ge 99.999 wt.%, Cr 99.5 wt.%, Hf 99.99% wt.% and Al 99.999% wt.%) to prepare the alloys. The elemental charges were placed in a copper water-cooled crucible and were melted in a high purity argon atmosphere using a tungsten electrode. Each alloy was melted five times to homogenize its composition as much as possible. A tube furnace (Lenton Furnaces, Market Harborough, UK) was used for the heat treatments. The specimens for the latter were cut from the bulk of the buttons, wrapped in Ta foil and placed in an alumina crucible. They were heat treated at 1400 °C for 100 h under an argon atmosphere. Pure Ti sponge was used in the tube furnace as oxygen getter.

The microstructures of the cast and heat-treated alloys were characterized using X-ray diffraction (XRD), scanning electron microscopy (SEM) with micro-analysis and electron microprobe analysis (EPMA). For XRD we used a Siemens D500 X-ray diffractometer (HiltonBrooks Ltd, Crew, UK) with CuK_α_ radiation (λ = 1.540562 Å), an acceleration voltage of 40 kV, a current of 30 mA and a step of 0.02 degrees per second. The phases were identified using ICDD (International Centre for Diffraction Data) database. The XRD experiments used bulk specimens and thus texture effects were likely. The lattice parameters of the Nb_ss_ were calculated using the Nelson-Riley extrapolation method to an accuracy of 0.1%. For imaging and micro-analysis we used a Camscan Mk2 SEM (Camscan Electron Optics Ltd., Cambridge, UK) equipped with an energy dispersive X-ray spectrometer (EDS) and a CAMECA SX-51 Electron Microprobe (EPMA) (CAMECA SAS, Gennevilliers Cedex, France) with high purity standards of Nb, Ti, Si, Ge, Cr, Hf and Al elements that were ground and polished to 1 μm finish. High purity Co was used for calibration prior to EDS analysis. An accelerating voltage of 20 kV was used and the electron beam size was 1 μm. The count rate for the calibration was controlled to be about 3000 cps by adjusting the probe current. The ZAF correction method was used. 

Specimens from the top, bulk and bottom of the buttons were used for micro-analysis. In each specimen we did large area (0.5 mm × 0.5 mm) analysis, point (phase) analysis and area analysis of eutectic microstructures. Large area analysis data was used to study macrosegregation, which is common in arc melted Nb-silicide based alloys [16]. At least 10 point analyses were performed on phases with size ≥ 5 μm and at least 5 large area analyses were taken from each of the top, bulk and bottom areas of the buttons to determine actual compositions. In the results section, the analysis data is given with the average, minimum and maximum values and the standard deviation. 

The macrohardness of the alloys was measured using a CV Instruments Vickers hardness tester (CV Instruments, Bowers Group, Camberley, UK) (430 AAT) with a load of 10 kg and holding time of 15 s. For each measurement we ensured that the typical alloy microstructure was sampled by the indenter. The microhardness of Nb_5_Si_3_ was measured using a Mitutoyo hardness machine with a load of 0.05 kg and holding time of 15 s. The microhardness of the Nb_ss_ was measured with a load of 0.025 kg and holding time of 15 s. At least 10 measurements were taken for the macrohardness of the alloys and the microhardness of the aforementioned phases. Each indent was measured twice. A gas pycnometer was used to measure the densities of the as cast alloys. We used the Zeiss KSRun version 3 high resolution imaging analysis software and the large area backscatter electron images that were used for large area analyses to measure the area fractions of phases. 

## 3. Results

### 3.1. Nb-24Ti-18Si-5Al-5Cr-5Ge (Alloy ZF6)

**Figure 1:**
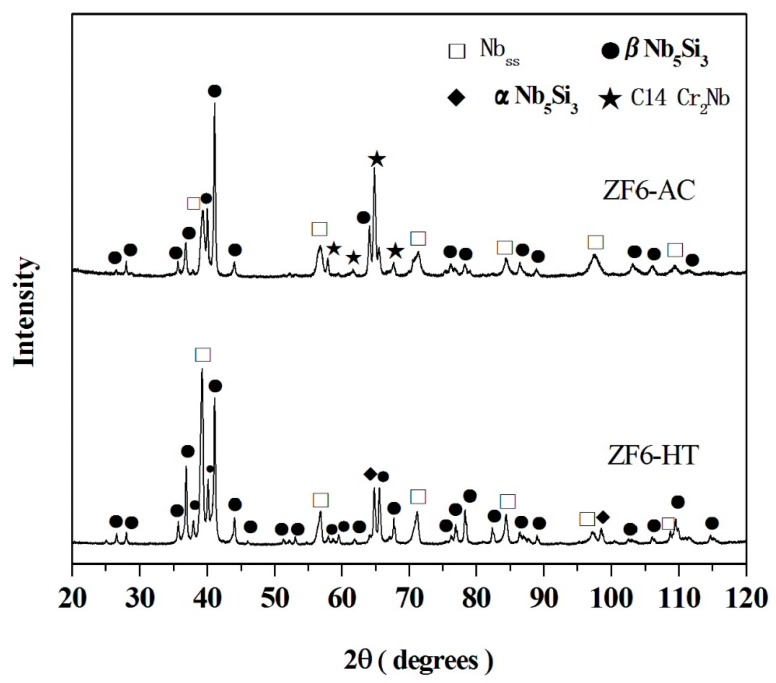
X-ray diffractograms of the as cast and heat treated (1400 °C/100 h) alloy ZF6.

**Figure 2:**
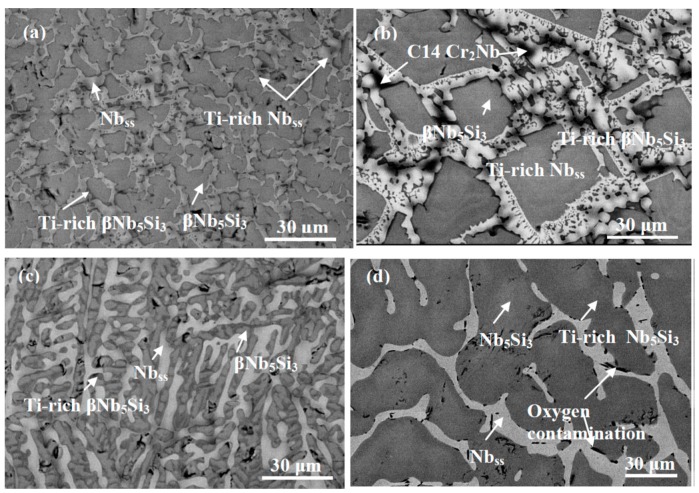
Typical backscatter electron images of the cross section of alloy ZF6: (**a**–**c**) top, bulk and bottom areas of the as cast alloy ZF6-AC; (**d**) the bulk of the heat-treated alloy ZF6-HT (1400 °C/100 h).

The actual composition of the as cast alloy (ZF6-AC) was Nb-25.7Ti-16.8Si-5.1Ge-5.0Cr-5.8Al and its density was 6.54 ± 0.01 g/cm^3^ (Table 1). There was macrosegregation of Si, Cr, Ti and Al the concentrations of which were in the range 15.8 to 20.1 at.%, 3.5 to 5.6 at.%, 23.9 to 26.3 at.% and 4.1 to 6.7 at.%, respectively (Table 2). The microstructure of ZF6-AC consisted of the Nb_ss_, βNb_5_Si_3_ and C14-Cr_2_Nb phases (Figure 1 and Table 2). Typical images of the microstructure of different parts of ZF6-AC are shown in Figure 2a–c. The βNb_5_Si_3_ grains in the bulk of ZF6-AC were large and faceted (Figure 2b). The Laves phase was formed between Ti-rich Nb_ss_ dendrites only in the bulk of the button (Figure 2b) where it was present at a small volume fraction and its average composition was 18.4Nb-24.8Ti-6.2Si-1.2Ge-46.3Cr-3.1Al (Table 2). The Nb_ss_ + βNb_5_Si_3_ eutectic was also observed in the top and bulk of ZF6-AC with average composition 45.5Nb-28.4Ti-8.1Si-3.7Ge-8.0Cr-6.3Al. Ti-rich areas were formed at the edges of Nb_ss_ and βNb_5_Si_3_. In the Ti-rich Nb_ss_ the concentrations of Si, Ge and Al were very close to those in the Nb_ss_ but the Cr concentration was higher (Table 2). The βNb_5_Si_3_ was leaner than the Si concentration in unalloyed Nb_5_Si_3_. The microstructure in the bottom of the button was different compared with the top and bulk and consisted only of the βNb_5_Si_3_ and Nb_ss_ phases (Figure 2c) with a higher volume fraction of the Nb_ss_ (Table 1). The microstructure shown in Figure 2c had evolved from a very fine eutectic that had formed at the very bottom of ZF6-AC where the melt had been in direct contact with the water-cooled copper crucible.

**Figure 3:**
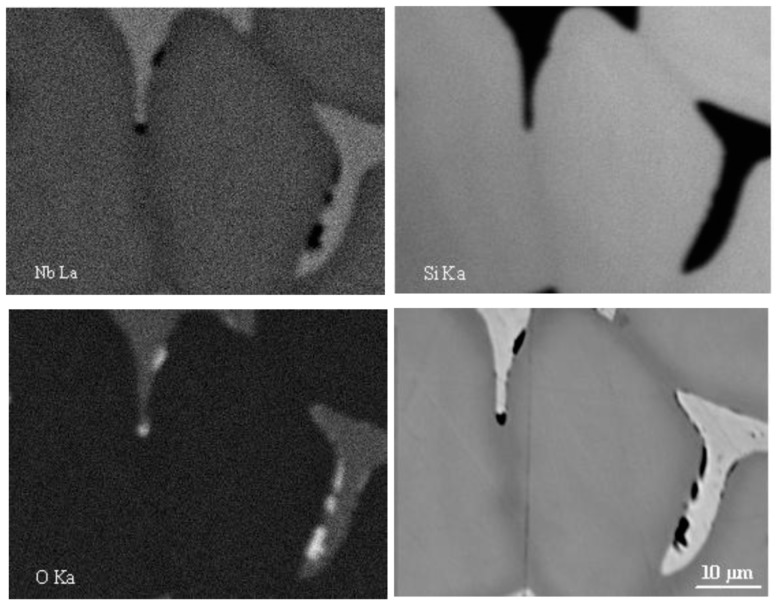
EPMA/Wavelength Dispersive spectroscopy (WDS) X-ray maps from the centre of the cross section of ZF6-HT (1400 °C/100 h) showing oxygen contamination at the edges of some of Nbss grains.

After the heat treatment the actual composition of the heat-treated specimen (ZF6-HT) was 43.1Nb-24.3Ti-17.4Si-5.8Ge-4.2Cr-5.2Al (Table 2). The XRD data (Figure 1) would suggest that the Nb_ss_, βNb_5_Si_3_ and αNb_5_Si_3_ phases were present. The microstructure is shown in Figure 2d. The volume fraction of the Nb_ss_ did not change significantly (Table 1). Ti-rich areas were still observed in the Nb_5_Si_3_. The C14-Cr_2_Nb Laves phase and the eutectic were absent after the heat treatment. A phase with black contrast was formed at a small volume fraction at the edges of some Nb_ss_ and Nb_5_Si_3_ grains. X-ray elemental maps taken in EPMA confirmed that this phase was rich in oxygen and Ti (Figure 3). 

### 3.2. Nb-24Ti-18Si-5Ge -5Cr-5Al-5Hf (Alloy ZF9)

**Figure 4:**
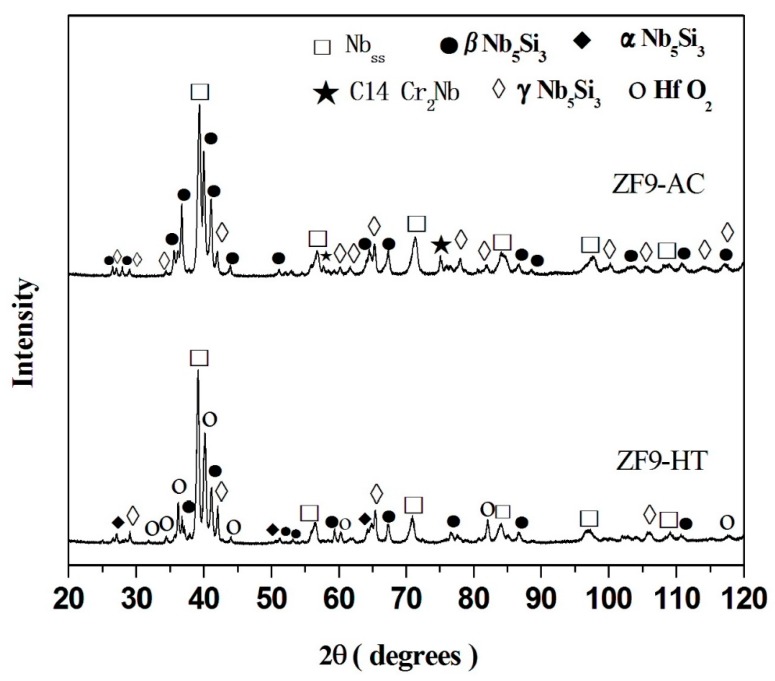
X-ray diffractograms of the as cast (AC) and heat treated (HT) (1400 °C/100 h) alloy ZF9.

**Figure 5:**
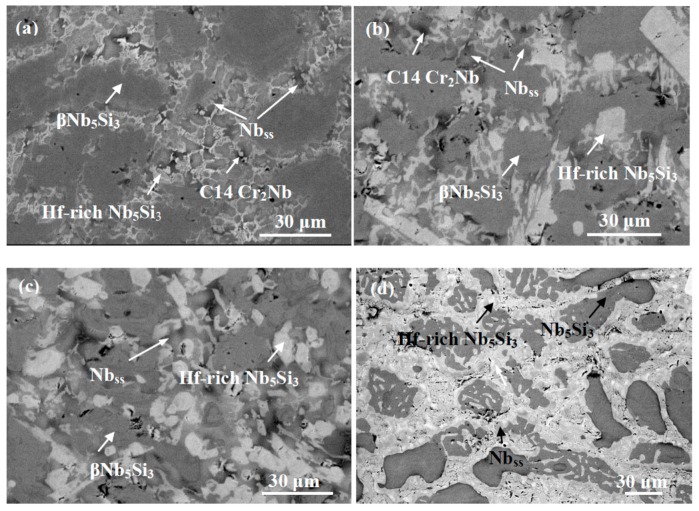

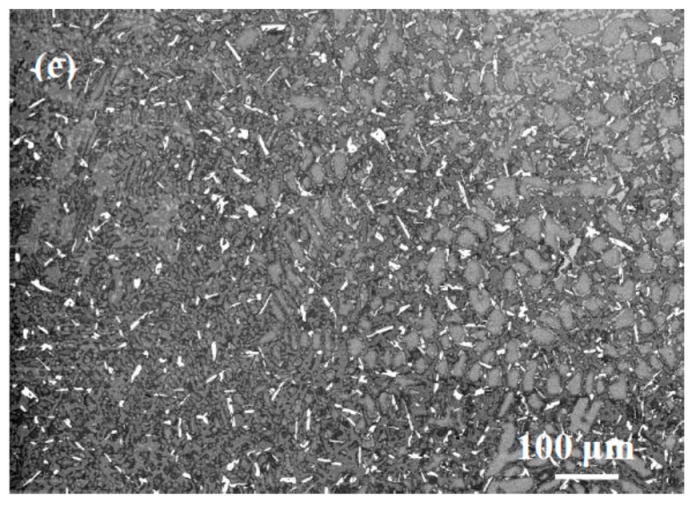
Typical backscatter electron images of the alloy ZF9: (**a**–**c**) top, bulk and bottom areas of the as cast alloy ZF9-AC; (**d**) the bulk area and (**e**) the top area of the heat-treated alloy ZF9-HT (1400 °C/100 h). In (**e**) the phase exhibiting white contrast is HfO_2_.

The actual composition of the as cast alloy (ZF9-AC) was 35.8Nb-26.4Ti-16.4Si-5.3Ge-5.6Cr-5.1Al-5.4Hf and its density was 6.96 ± 0.02 g/cm^3^ (Table 1). There was macrosegregation of Ti, Si, Al and Cr the concentrations of which were in the ranges 24.1 to 27.5 at.%, 15.3 to 18.4 at.%, 4.1 to 6.1 at.% and 4.3 to 6.4 at.%, respectively (Table 3). The phases present in the microstructure of ZF9-AC were the Nb_ss_, βNb_5_Si_3_, γNb_5_Si_3_ and C14-Cr_2_Nb (Figure 4 and Table 3). Typical images of the microstructure are shown in Figure 5a–c. The partitioning of Hf between the aforementioned phases made the characterisation of the microstructure extremely difficult owing to only slight variations in contrast between different phases under back scatter electron imaging conditions. The phase with grey contrast in Figure 5 was Nb_5_Si_3_ with average composition 37.2Nb-21.0Ti-24.2Si-6.6Ge-1.2Cr-5.0Al-4.8Hf and Si + Ge + Al concentration about 35.8 at.%, close to the Si concentration in unalloyed Nb_5_Si_3_. The phase with light grey contrast in Figure 5 was Hf-rich Nb_5_Si_3_ in which the Hf concentration was almost double that in the Nb_5_Si_3_. The Nb_ss_ was formed between the former two phases and exhibited dark gray contrast. Reliable results of the volume fraction of the Nb_ss_ in this alloy could not be obtained as the contrast of the solid solution was close to that of the Nb_5_Si_3_ and Hf-rich Nb_5_Si_3_. The C14-NbCr_2_ Laves phase was formed between the Nb_ss_ and Hf-rich Nb_5_Si_3_ in the top and bulk of ZF9-AC and its average composition was 20.5Nb-19.7Ti-5.6Si-1.3Ge-43.7Cr-4.0Al-5.2Hf. As was the case in the alloys Nb-24Ti-18Si-5Cr-5Ge [15] and ZF6, the Laves phase was absent in the bottom of the button of ZF9-AC. The Nb_ss_ + Nb_5_Si_3_ eutectic was not observed in any part of the button of this alloy. A microstructure exhibiting fine features that could be representative of eutectic was observed only once in one area in the top of ZF9-AC. Detailed study of the button did not find any other areas with similar features.

After the heat treatment the actual composition of the heat-treated specimen (ZF9-HT) was 38.0Nb-24.2Ti-17.1Si-5.2Ge-4.8Cr-5.5Al-5.2Hf (Table 3). The XRD data (Figure 4) would suggest that the phases Nb_ss_, βNb_5_Si_3_, γNb_5_Si_3_, αNb_5_Si_3_ and HfO_2_ were present. The C14-NbCr_2_ Laves phase was absent. The microstructure is shown in Figure 5d. The Hf-rich Nb_5_Si_3_ was still observed. Hafnia particles exhibiting white contrast were found only near the surface of ZF9-HT (Figure 5e). Their average composition (EPMA/WDS) was 34.7Hf-65.3O.

The lattice parameter of the Nb_ss_ in the alloys ZF6 and ZF9, and hardness data are given in the Table 4 and Table 5, respectively. The lattice parameter of Nb_ss_ increased after the heat treatment. The addition of Hf in the alloy ZF9 caused a decrease in hardness compared with the alloy ZF6.

## 4. Discussion

### 4.1. Macrosegregation

Macrosegregation is a common phenomenon in as cast Nb-silicide based alloys that are produced in water cooled copper crucibles using non-consumable W electrodes for arc melting [16]. It is also observed in Nb-silicide based alloys that are prepared using vacuum induction melting and induction skull melting [17]. 

In the alloy ZF6 there was macrosegregation of Si. The latter was low when Ge was in synergy only with Si and increased after alloying with Ti (see Table 6 and compare alloys ZF1 [9] and ZF3 [18]) and with Ti, Al and Cr (Table 6, alloy ZF6). In the alloy ZF9, the addition of Hf reduced the macrosegregation of Si and Al (Table 6 and Table 7), increased that of Ti (Table 7) and did not change the macrosegregation of Cr (Table 7).

Long range transport due to the flow of melt and movement of solid during solidification play a key role in the formation of macrosegregation [19]. The latter also is affected by the composition of the alloy and the viscosity of its melt [19]. Complex interrelationships of heat transfer, solute transport, solid movement and fluid flow control macrosegregation [16]. The macrosegregation of Si in Nb-silicide based alloys has been attributed to such interrelationships that affect the buoyancy and thermo-capillary forces that arise by solute partitioning in these alloys and their effects on the densities of phases and the sign and value of dγ_L_/dT (γ_L_ is the liquid surface tension) [16].

**Figure 6:**
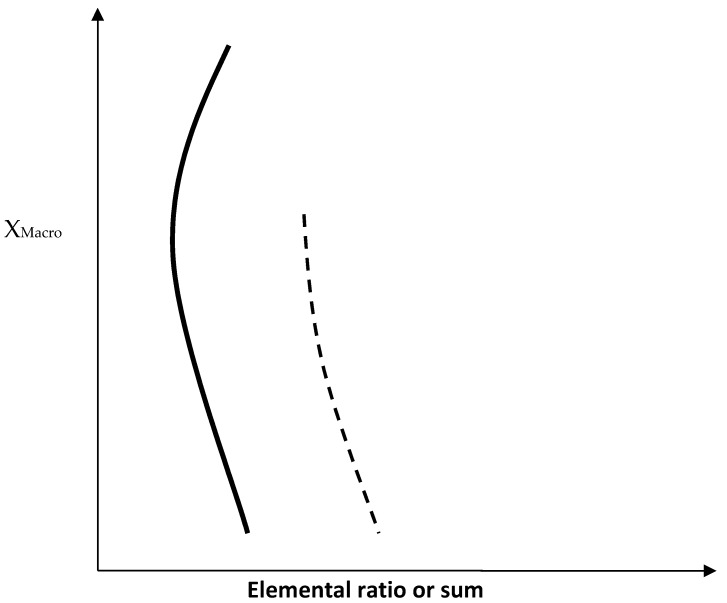
Schematic diagram showing the dependence of the macrosegregation of element X (vertical axis) on the alloying elements in specific phases in Nb silicide-based alloys. The ranges of X values are given in Table 6 and Table 7. Chemical analysis data for the Nb_ss_ + Nb_5_Si_3_ eutectic in Ge containing Nb silicide based alloys is given in the Table 8 and Table 9. The chemical analysis data for specific phases in Nb silicide based alloys can be found in the references given in Table 6, Table 7, Table 8 and Table 9. The thick line shows (a) how the Si macrosegregation varies with (Si + Ge)_eutectic_ (range 11.8 to 17.7, nose ZF6), (Si/Al)_Nb5Si3_ (range 4.8 to 9.5, nose ZF9), (Si/Cr)_Nb5Si3_ (range 16.6 to 24, nose ZF6), (Si/Al)_Nbss_ (range 0.21 to 0.49, nose ZF6), (TM/SM)_Nbss_ (range 2.7 to 3.8, nose ZF5), (TM/SM)_Ti rich Nbss_ (range 3.4 to 5.6, nose ZF5), (Si/Cr)_Nbss_ (range 0.1 to 0.28, nose ZF9) and (Ti/Cr)_Nbss_ (range 2.1 to 3.6, nose ZF9), (b) how the Ti macrosegregation varies with (Si/Cr)_Nb5Si3_ (range 16.6 to 24, nose ZF6) and (Si/Al)_Ti rich Nb5Si3_ (range 4.4 to 8.7, nose ZF6), (Ti/Cr)_Nb5Si3_ (range 14.9 to 17.5, nose ZF4) and (Ti/Cr)_Ti rich Nb5Si3_ (range 9.8 to 30, nose ZF4) and (c) how the Cr macrosegregation varies with (Si/Cr)_Nbss_ (range 0.1 to 0.28, nose ZF9). X = Si, Ti, Cr and TM = Ti + Cr + Hf, SM = Si + Ge + Al. The dashed line shows (d) how the macrosegregation of Si varies with (Si/Al)_Ti rich Nb5Si3_ (range 4.3 to 8.7) and (Si + Al + Ge)_eutectic_ (range 18.1 to 22.6) and (e) how the Ti macrosegregation varies with (Si/Al)_Nb5Si3_ (range 4.8 to 9.5) and (Si/Al)_Nbss_ (range 0.21 to 0.33), X = Si, Ti.

**Figure 7:**
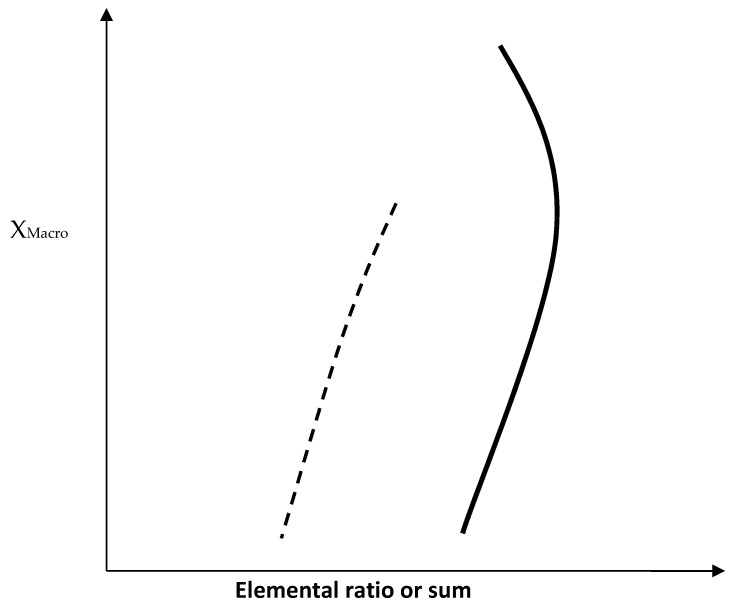
Schematic diagram showing the dependence of the macrosegregation of element X (vertical axis) on alloying elements in specific phases in Nb silicide-based alloys. The ranges of X values are given in Table 6 and Table 7. The chemical analysis data for specific phases in Nb silicide-based alloys can be found in the references given in the Table 6, Table 7, Table 8 and Table 9. The thick line shows (a) how the Si macrosegregation varies with ([Si + Ge]/Cr)_Laves_ (range 2.3 to 4.4, nose ZF6), (TM/[Si + Ge])_Nbss_ (range 6.8 to 18.9, nose ZF9), (Ti/Cr)_Nb5Si3_ (range 12.7 to 17.5, nose ZF9), (Ti/Cr)_Ti rich Nb5Si3_ (range 9.8 to 30, nose ZF9), (TM/SM)_Nb5Si3_ (range 0.5 to 0.8, nose ZF9), (TM/SM)_Ti rich Nb5Si3_ (range 0.6 to 1, nose ZF9) and (TM/[Si + Ge])_Nb5Si3_ (range 0.6 to 0.9, nose ZF9) (b) how the Ti macrosegregation varies with (Si/Cr)_Nbss_ (range 0.1 to 0.3, nose ZF4) and (Ti/Cr)_Nbss_ (range 2.1 to 3.6, nose ZF4) and (c) how the Cr macrosegregation varies with (Si/Cr)_Nb5Si3_ (range 16.6 to 20.2, nose ZF9). X = Si, Ti, Cr, TM = Ti + Cr + Hf and SM = Si + Ge + Al. The dashed line shows (d) how the macrosegregation of Si varies with ([Si + Ge + Al]/Cr)_Laves_ (range 1.4 to 5.5) and (TM/([Si + Ge])_Ti rich Nbss_ (range 9 to 13.2) and (e) how the Ti macrosegregation varies with (Ti/Cr)_Ti rich Nbss_ (range 2.1 to 2.6). X = Si, Ti and TM = Ti + Cr + Hf.

The effect of the synergy of Ge with Hf and other alloying elements on the macrosegregation of Si, Ti and Cr can be understood by studying the role played by solute partitioning in the solidification of Nb-silicide based alloys. The chemical analysis data for the phases present in the alloys for which macrosegregation data is summarised in the Table 6 and Table 7 can help one to find out whether there is dependence of macrosegregation on elemental ratios or sums in the Nb_ss_, Nb_5_Si_3_, NbCr_2_ phases and the Nb_ss_ + Nb_5_Si_3_ eutectic. Trends for the Si + Ge + Al and Si + Ge concentrations in Nb_ss_ + βNb_5_Si_3_ eutectics are shown in the Table 8 and Table 9, respectively. The Figure 6 and Figure 7 show the trends in the macro-segregation of Si and Ti for different elemental ratios and sums.

The Figure 6 and Figure 7 show that Si macrosegregation in Nb-silicide based alloys is linked with solute partitioning during solidification, as evidenced by the variation of Si macrosegregation with the ratios and sums of different elements. Figure 6 shows that the ratios (Si/Al)_Nbss_, (Si/Cr)_Nbss_, (Ti/Cr)_Nbss_, (TM/SM)_Nbss_, (TM/SM)_Ti rich Nbss_ (where TM = Ti + Cr + Hf and SM = Si + Ge + Al), (Si/Al)_Nb5Si3_, (Si/Cr)_Nb5Si3_, and the (Si+Ge)_eutrectic_ all exhibit the same trend with increasing Si macrosegregation. The “nose” of the curves corresponds to the alloys ZF6 or ZF9. In Figure 6 the trend of the (Si/Al)_Ti rich Nb5Si3_ and (Si + Al + Ge)_eutectic_ is shown by the lower part of the curves. The trend in Figure 6 is opposite to the trend shown in Figure 7 for the ratios (Ti/Cr)_Nb5Si3_, (Ti/Cr)_Ti rich Nb5Si3_, (TM/SM)_Nb5Si3_, (TM/SM)_Ti rich Nb5Si3_, (TM/([Si + Ge])_Nb5Si3_ (where TM = Ti + Cr + Hf and SM = Si + Ge + Al) and (TM/[Si + Ge])_Nbss_, ([Si + Ge]/Cr)_Laves_ where the “nose” corresponds to the alloy ZF9. The trend of ([Si + Ge + Al]/Cr)_Laves_ and (TM/([Si + Ge])_Ti rich Nbss_ in Figure 7 is shown by the lower part of the curves and is opposite to that shown in Figure 6. The data would thus suggest that Hf in synergy with Ge, Al, Cr and Ti can reduce, but not eliminate, the macrosegregation of Si and this can be attributed to solute partitioning leading to “maxima” or minima” is specific elemental ratios and sums in the key phases that are present in these alloys. It should be noted that all the aforementioned elements are required for achieving a balance between environmental and mechanical properties in Nb-silicide based alloys [3].

The Figure 6 and Figure 7 also show that Ti macrosegregation in Nb-silicide based alloys is linked with solute partitioning during solidification as evidenced by the variation of Ti macrosegregation with different element ratios and sums. The Figure 6 shows that the ratios (Si/Cr)_Nb5Si3_, (Si/Al)_Ti rich Nb5Si3_, (Ti/Cr)_Nb5Si3_ and (Ti/Cr)_Ti rich Nb5Si3_ exhibit the same trend with increasing Ti macrosegregation. The “nose” of the curves corresponds to the alloys ZF6 or ZF4. In Figure 6 the trend of (Si/Al)_Nb5Si3_ and (Si/Al)_Nbss_ is shown by the lower part of the curves. The trend in Figure 6 is opposite to the trend shown in Figure 7 for the ratios (Si/Cr)_Nbss_ and (Ti/Cr)_Nbss_ where the “nose” corresponds to the alloy ZF4. The trend of (Ti/Cr)_Ti rich Nbss_ in Figure 7 is shown by the lower part of the figure and is opposite to that shown in Figure 6. The data would thus suggest that in Ge containing Nb-silicide based alloys the Cr plays the important role in the macrosegregation of Ti owing to partitioning between the Nb_ss_ and the Nb_5_Si_3_ silicide. 

Figure 6 and Figure 7 also show that Cr macrosegregation in Nb-silicide based alloys is linked with solute partitioning during solidification as evidenced by the variation of Cr macrosegregation with different elemental ratios and sums. These two figures show that the ratios (Si/Cr)_Nb5Si3_ and (Si/Cr)_Nbss_ exhibit opposite trends with the “nose” of the curves corresponding to the alloy ZF9. This would suggest that Hf plays a role in the macro-segregation of Cr. The latter is not strong in Nb-silicide based alloys with Cr ≤ 8 at.% compared with Si or Ti (see Table 7). The Hf is also known not to affect significantly the partitioning of Cr between the Nb_ss_ and the Nb_5_Si_3_ silicide. Thus, the effect of the synergy of Ge and Hf on the macrosegregation of Cr is expected to be weak.

### 4.2. Microstructures

#### 4.2.1. Cast Alloys

In both alloys the Nb_5_Si_3_ was the primary phase. The solidification paths in the bulk of the ingots were L → L + βNb_5_Si_3_ → L + βNb_5_Si_3_ + (Nb_ss_ + βNb_5_Si_3_) _eutectic_ → L + βNb_5_Si_3_ + (Nb_ss_ + βNb_5_Si_3_) _eutectic_ + C14-NbCr_2_ and L → L+ Nb_5_Si_3_ → L + Nb_5_Si_3_ + Nb_ss_ → L + Nb_5_Si_3_ + Nb_ss_ + C14-NbCr_2_, respectively for the alloys ZF6 and ZF9 with the C14-NbCr_2_ forming from the last to solidify liquid. The addition of Hf suppressed the Nb_ss_ + βNb_5_Si_3_ eutectic but not the Laves phase. 

The formation of the Laves phase is linked with Cr redistribution during solidification [12]. This would explain its absence in the bottom of the buttons owing to the effect of cooling rate on Cr partitioning [14,15]. The addition of Hf made possible the formation of the C14-NbCr_2_ Laves phase at higher cooling rates in ZF9-AC, compared with ZF6-AC.

Compared with the alloy KZ5-AC (Nb-24Ti-18Si-5Al-5Cr [12]) the addition of Ge in ZF6-AC decreased the solid solubility of Cr in the Nb_ss_. Compared with the alloy ZF6-AC the addition of Hf in ZF9-AC (i) increased the solid solubilities of Cr and Al and decreased the solid solubility of Ge in the Nb_ss_. 

There are three types of 5-3 silicides in the binary Nb-Si system [20], namely the βNb_5_Si_3_ (t*I*32, I4/mcm, prototype W_5_Si_3_), αNb_5_Si_3_ (t*I*32, I4/mcm, prototype Cr_5_B_3_) and γNb_5_Si_3_ (h*P*16, P6_3_/mcm, prototype Mn_5_Si_3_), the former two are the equilibrium phases and the third is metastable and is stabilised by interstitial impurities [20]. The latter silicide has the same structure with the Ti_5_Si_3_ and Hf_5_Si_3_ [21] silicides. Thus, it is expected that alloying the Nb_5_Si_3_ with Ti or/and Hf would tend to increase the stability of the γNb_5_Si_3._ The XRD data of ZF9-AC (Figure 4) contained characteristic peaks corresponding to the γNb_5_Si_3_. Bewlay et al [22] suggested that the Nb_5_Si_3_ had hexagonal rather than tetragonal structure when the Nb/(Ti+Hf) ratio was less than one in the alloy and silicide. For the Nb_5_Si_3_ and Hf rich Nb_5_Si_3_ silicides in the present work the above ratio was in the range 1.4 to 1.5 (Table 3). 

The Al content of the Nb_ss_ + βNb_5_Si_3_ eutectic in the alloy ZF6 (Table 8) was the same with the eutectic in the cast alloy ZF5 (Nb-24Ti-18Si-5Al-5Ge [13]) but not the Si + Ge content (Table 9). This was attributed to the synergy of Al and Ti, which is stronger than that of Cr and Ti (Table 9, compare the alloys ZF8-AC and ZF5-AC with the alloys ZF7-AC and ZF4-AC). 

The C14-NbCr_2_ Laves phase was observed in the bulk of the buttons of the alloys ZF6-AC, ZF9-AC and in ZF4 (Nb-24Ti-18Si-5Cr-5Ge [15]), and was absent in the bottom of the buttons of all three alloys but present in the top only of the latter two alloys. If we were to use the ratio Λ = [Si + Ge (+ Al)]/Cr as a criterion for the sensitivity of C14-NbCr_2_ Laves phase formation on alloy composition and consider the large area analysis data for the top, bulk and bottom of the above alloys given in Table 2 and Table 3 and [15], it is concluded that the lowest value of Λ for Laves phase formation in Ge containing alloys is about 3.7 at.% when Al is not present in the alloy and about 4.3 at.% when Al is in synergy with Cr. 

In the alloys ZF6-AC and ZF9-AC the Laves phase was the hexagonal C14-Cr_2_Nb (h*P*12) (Figure 1 and Figure 4). In the Laves phase the Al and Si atoms substitute for Cr atoms [23]. Furthermore, alloying with Si [24] or Al [25] stabilises the C14-NbCr_2_ to low temperatures. The Si + Ge + Al + Cr concentration in the Laves phase (or B element content in AB_2_ = NbCr_2_) in the alloys ZF6 and ZF9 was respectively 56.8 at.% and 54.6 at.% and the Ge concentrations were 1.2 at.% and 1.3 at.%, respectively. Thus, the B element content in the C14-NbCr_2_ was similar to that reported in the alloys KZ4 (Nb-24Ti-18Si-5Cr) and KZ7 (Nb-24Ti-18Si-5Al) [12] and JG3 (Nb-24Ti-18Si-5Al-5Cr-5Hf-2Mo [26]) where the (Cr + Si + X)/(Nb + Y) ratio (X = Al, Y = Hf, Ti) was in the range 1.1 to 1.5 compared with (Cr + Si + X)/(Nb + Y) ratios of 1.3 and 1.2, respectively for the alloys ZF6 and ZF9 (X = Al, Ge, Y = Hf, Ti). The off-stoichiometric and lean in B element content NbCr_2_ has also been reported in alloys of the Nb-Cr-Ti [27] and Nb-Cr-Hf [28] ternary systems and was attributed to the anti-site defect substitution with Ti or Hf partitioning to A site and thus displacing some Nb atoms to the B site in AB_2_ = NbCr_2_. The Ge concentration in the Laves phase was in agreement with [10]. Considering the data for the C14-NbCr_2_ Laves phase in [15] it is suggested (a) that Al does not affect the solid solubility of Ge in this phase, which is about 1.3 at.% and (b) that in the presence of Ge the solid solubility of Si in the Laves phase is in the range 5.5 to 6.5 at.%.

#### 4.2.2. Heat Treated Alloys

After the heat treatment no Ti rich areas were observed in the Nb_ss_, meaning the latter was “homogenized”. The Si and Ge concentrations in the Nb_ss_ were low (Table 2 and Table 3) and in agreement with those in the binary Nb-Si and Nb-Ge systems [20,21]. Thus, the synergy of Cr and Al with/out Hf did not change significantly the solid solubilities of both Si and Ge in the Nb_ss_ and did not affect the “homogenization” of the solid solution. In the Nb_ss_ in ZF6-HT the solid solubilities of Cr and Al did not change but in ZF9-HT the solid solubility of both Cr and Al decreased. This was attributed to the synergy of Hf and Ge. The Laves phase was absent in both alloys, as was also the case in the heat-treated alloys KZ5 (Nb-24Ti-18Si-5Al-5Cr [12]) and JG3 (Nb-24Ti-5Al-5Cr-5Hf-2Mo [26]). 

According to the XRD data (Figure 1 and Figure 4) the βNb_5_Si_3_ had partially transformed to the αNb_5_Si_3_ in both alloys. Thus, equilibrium was not reached in both alloys after 100 h at 1400 °C. This and the data in [13,15,18] for heat treated Ti and Ge containing alloys would suggest that alloying with Ge makes the transformation of βNb_5_Si_3_ to αNb_5_Si_3_ more sluggish. 

In the Nb_5_Si_3_ the solid solubilities of Ti, Si, Cr, Al and Ge were essentially the same in both alloys. The Hf-rich areas in Nb_5_Si_3_ were still present in ZF9-HT with no significant change in the solubility of Hf. Compared with the data for Hf containing Nb-silicide based alloys in [29,30] it is concluded that the solubility of Hf in Nb_5_Si_3_ is not affected by the presence of Ge in the alloy.

In the alloy ZF6-HT, a very small volume fraction of Ti rich oxide was formed at the edges of some of its Nb_ss_ and Nb_5_Si_3_ grains (Figure 2d and Figure 3). This oxide was also observed in heat treated Ti and Ge containing alloys with no Hf present [13,15,18], but was absent in Ti free but Ge containing heat treated Nb-silicide based alloys [9,13,18]. This phase formed because of the contamination of the alloy by O during heat treatment. In the ZF9-HT, HfO_2_ was formed instead of titanium oxide. This is in agreement with data for heat treated Hf and Ti-containing Nb-silicide based alloys [29,30] and confirmed the scavenging of oxygen by Hf.

#### 4.2.3. Lattice Parameter of The Nb_ss_

The atomic radii of Nb, Ti, Si, Ge, Cr, Al and Hf are 1.47 Å, 1.47 Å, 1.17 Å, 1.39 Å, 1.30 Å, 1.43 Å and 1.59 Å, respectively [31]. Compared with the alloys ZF4 (Nb-24Ti-18Si-5Cr-5Ge [15]) and ZF5 (Nb-24Ti-18Si-5Al-5Ge [13]), the lattice parameter of the Nb_ss_ in the alloy ZF6 respectively decreased by about 0.40% and 0.55% in the as cast condition, and by about 0.28% and 0.73% in the heat treated condition (Table 4). The contraction of the lattice of Nb_ss_ individually by Cr and Al was discussed in [13,15]. The data for the alloy ZF6 would thus suggest that the synergy of Ge with Al and Cr led to further contraction of the Nb_ss_ lattice. The increase of the lattice parameter of the Nb_ss_ in ZF6-HT was attributed to the reduction in the concentrations of Ti, Si, Ge and Al.

The addition of Hf to the Nb_ss_ was reported to increase the lattice parameter owing to the larger atomic radius of Hf compared with that of Nb [32,33]. In this work the lattice parameter of the Nb_ss_ in the alloy ZF9 was 0.12% higher than that in the alloy ZF6 in the as cast and heat-treated conditions. The increase of the Nb_ss_ lattice parameter in ZF9-HT, compared with the as cast condition, was attributed to the reduction of the concentrations of all solute elements.

### 4.3. Comparison with High Entropy Alloys

Some Nb-silicide based alloys, some bcc Nb solid solutions in Nb-silicide based alloys and some of the eutectics with bcc Nb_ss_ + βNb_5_Si_3_ in Nb-silicide based alloys meet the “accepted” definition of High Entropy Alloys (HEAs) [3]. According to this definition, which asserts that HEAs have principal elements with the concentration of each element being between 35 and 5 at.% [34], the alloy ZF9 is a HEA. However, the alloy ZF6 does not comply with this description of HEAs. More specifically, the alloy ZF9 is a refractory metal (RM) based bcc solid solution + intermetallics HEA or a RM complex concentrated alloy (CCA). The parameters ΔH^chem^, ΔS_mix_, VEC, δ, Δχ and Ω have been considered for solid solution(s) and solid solution(s) + intermetallic(s) HEAs [34] and Nb-silicide based alloys [3,35,36]. The data in Table 10 shows that the above parameters of the alloys ZF6 and ZF9 do not differ significantly, even though only one of them (ZF9) meets the “criterion” for HEAs. The data in Table 10 shows that the solid solutions of both alloys also do not comply with the above definition of HEAs. The aforementioned parameters of both alloys and their solid solutions are in the ranges for Nb-silicide based alloys [35] and their solid solutions [36]. 

Research on HEAs has indicated ranges for the above parameters for bcc solid solution(s) and bcc solid solution(s) + intermetallic(s) HEAs. Both alloys of this study have ΔH^chem^ more negative than bcc solid solution + intermetallic HEAs, their parameters ΔS_mix_ and δ are in the range of bcc solid solution + intermetallic HEAs, and the parameters VEC and Ω are smaller than those of bcc solid solution + intermetallic HEAs [35]. The bcc solid solutions in the alloys ZF6 and ZF9 (a) have ΔH^chem^ and ΔS_mix_, δ and Ω in the range of RM based bcc solid solution HEAs [37], (b) their Ω values fall in the lower range of Ω values for the RM based bcc solid solution HEAs [37] and (c) their parameters VEC, δ, Ω, Δχ and ΔH are in the range of bcc solid solution HEAs [38,39,40]. 

The density of RM HEAs (or RM CCAs) is in the range 5.59 g/cm^3^ (for bcc AlNbTiV) to 13.75 g/cm^3^ (for bcc MoNbTaW) [34]. The majority (74%) of the twenty-three RM HEAs reported in [34] have densities higher than those of the alloys ZF6 and ZF9. More specifically, only the NbTiV_2_Zr (bcc, 6.38 g/cm^3^) and CrNbTiVZr (bcc + Laves, 6.52 g/cm^3^) HEAs have densities lower than the alloy ZF6 and the latter alloys and the NbTiVZr (bcc, 6.5 g/cm^3^), CrNbTiZr (bcc + Laves, 6.7 g/cm^3^) and AlNb_1.5_Ta_0.5_Ti_1.5_Zr_0.5_ (bcc, 6.88 g/cm^3^) HEAs have densities lower than the alloy ZF9.

The hardness of RM bcc HEAs or CCAs is in the range 306 HV (NbTiV_2_Zr, bcc) to 591 HV (AlMo_0.5_NbTa_0.5_TiZr, bcc + B2) [34]. The hardness of the Nb_ss_ in ZF6-HT was higher than the aforementioned range and higher than that of the bcc solid solution RM based HEAs AlNb_1.5_Ta_0.5_Ti_1.5_Zr_0.5_, Al_0.4_Hf_0.6_NbTaTiZr, and Al_0.3_NbTa_0.8_Ti_1.4_V_0.2_Zr_1.3_ reported in [37].

### 4.4. Hardness

The hardness of the alloy ZF6 was higher by 11.9% and 8.4% compared with the alloy ZF4 (Nb-24Ti-18Si-5Al-5Ge [13]), and lower by 3.6% and 8.2% compared with the alloy ZF5 (Nb-24Ti-18Si-5Cr-5Ge [15]) in the as cast and heat treated conditions, respectively. The alloy ZF6 exhibited good retention of its hardness after the heat treatment at 1400 °C, as was the case for the alloys ZF4 and ZF5. The hardness data for these three alloys would suggest that in the synergy of Ge with Al and Cr the latter is the key element. Compared with the alloy ZF6, the addition of Hf in the alloy ZF9 resulted to a reduction of hardness by 4.6% and 8.8% in the as cast and heat-treated conditions, respectively. The alloy ZF9 exhibited good retention of its hardness after the heat treatment as the hardness was lower by 2.7% than that of ZF9-AC. 

The synergy of Cr and Al in the alloy ZF6 cancelled out the negative effect of Al on the hardness of Nb_ss_ [13,15] and would suggest that the synergy of Cr and Ge is key to strengthening the Nb_ss_. The hardness of Nb_5_Si_3_ in the alloy ZF6 was almost the same as in ZF4 [13] in both the as cast and heat-treated conditions, and lower than that in the alloy ZF5 [15]. This would suggest that in the synergy of Al and Cr with Ge the Al “controls” the hardness of Nb_5_Si_3_. The hardness of Nb_5_Si_3_ in ZF9 was lower in both the as cast and heat-treated conditions compared with ZF6. This would suggest that the synergy of Hf with Al, Cr and Ge has a softening effect on Nb_5_Si_3_. The hardness of Nb_5_Si_3_ in ZF9-HT was the same as that of unalloyed Nb_5_Si_3_.

The application of the rule of mixtures (ROM) to calculate the strength of composites assumes the matrix of the composite to be unaffected by the reinforcement. The hardness of ZF6-HT calculated using ROM is 1423 HV and the measured value was 854 HV (Table 5). In the Ti free alloys ZF1 (Nb-18Si-5Ge) and ZF2 (Nb-18Si-10Ge) with microstructures consisting of Nb_ss_ and Nb_5_Si_3_, the same as ZF6-HT, best agreement with the experimental value was given by a Pythagorean type addition rule [9]. If we assume that the Pythagorean rule [41] applies in the case of ZF6-HT a correction of 0.652 would be necessary to match the two values. This correction is essentially the same with the average correction of 0.638 for all alternative addition rules of calculating HV [9,42].

The hardness of the alloys ZF1-HT15 and ZF2-HT15 respectively was 812 HV and 819 HV and the microhardness of Nb_5_Si_3_ in the two alloys was 1532 and 1526 HV, respectively [9]. In other words, the hardness values of these alloys and their Nb_5_Si_3_ silicides were not significantly different than those of the alloy ZF6-HT. Why the Pythagorean rule addition did not work in the case ZF6-HT? The alloys ZF6 and ZF2 essentially were intermetallic matrix composites, ZF6 is different from ZF2 (see below) and ZF1 is a metal matrix composite. We would like to suggest that the difference is attributed to the microstructures of the three alloys and in particular the constraint effects on hardness.

The strength of in-situ composites is strongly affected by the size, shape and distribution of the matrix and reinforcement phase(s). The presence of sharp corners in the latter, which can act as stress concentrators, must be taken into account in composite strengthening [43]. The constraint imposed by the brittle intermetallic phase on the ductile matrix can contribute significantly to the composite strength. Research on composites has considered different strengthening mechanisms which either independently or simultaneously can be responsible for the strength of a composite. The proposed mechanisms include (a) strengthening due to constrained plastic flow in the ductile matrix owing to the presence of brittle intermetallics [44] and (b) cavitation at the interfaces and the matrix in the high stressed regions near sharp corners in the reinforcement [45]. 

The microstructure of the as cast alloy ZF6 had a very high (>0.8) volume fraction of strongly faceted large Nb_5_Si_3_ grains with thinner layers of Nb_ss_ and Nb_ss_ + Nb_5_Si_3_ eutectic confined in between the Nb_5_Si_3_ grains (Figure 2b). After the heat treatment the prior eutectic was not observed in between the large silicide grains. The as cast alloys ZF1 and ZF2 had microstructures consisting of primary Nb_5_Si_3_ and Nb_ss_ + Nb_5_Si_3_ eutectic. The vol.% of primary Nb_5_Si_3_ was slightly more than double in the alloy ZF2 [9]. In both alloys the primary Nb_5_Si_3_ was faceted. The ZF1-HT15 had Nb_5_Si_3_ with fine prior eutectic in between Nb_5_Si_3_ grains (constraint different from ZF6-HT), the ZF2-HT15 had similar microstructure with ZF1-HT5 but with higher vol.% of Nb_5_Si_3_ and less fine prior eutectic between the non-facetted Nb_5_Si_3_ grains (constraint different from ZF6-HT). 

**Figure 8:**
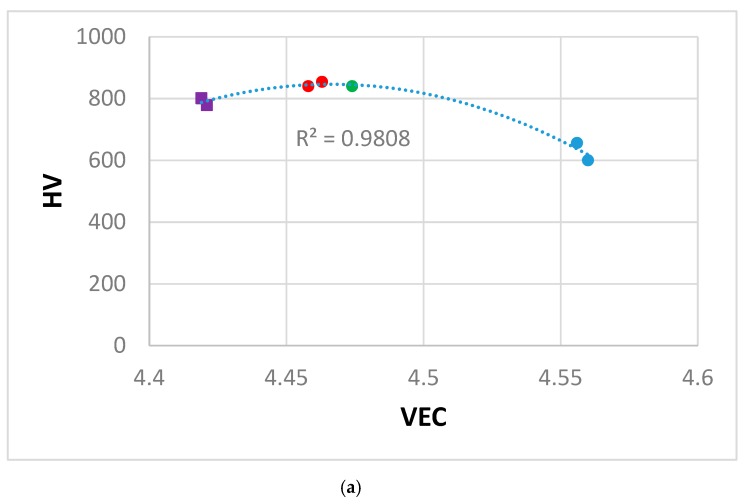

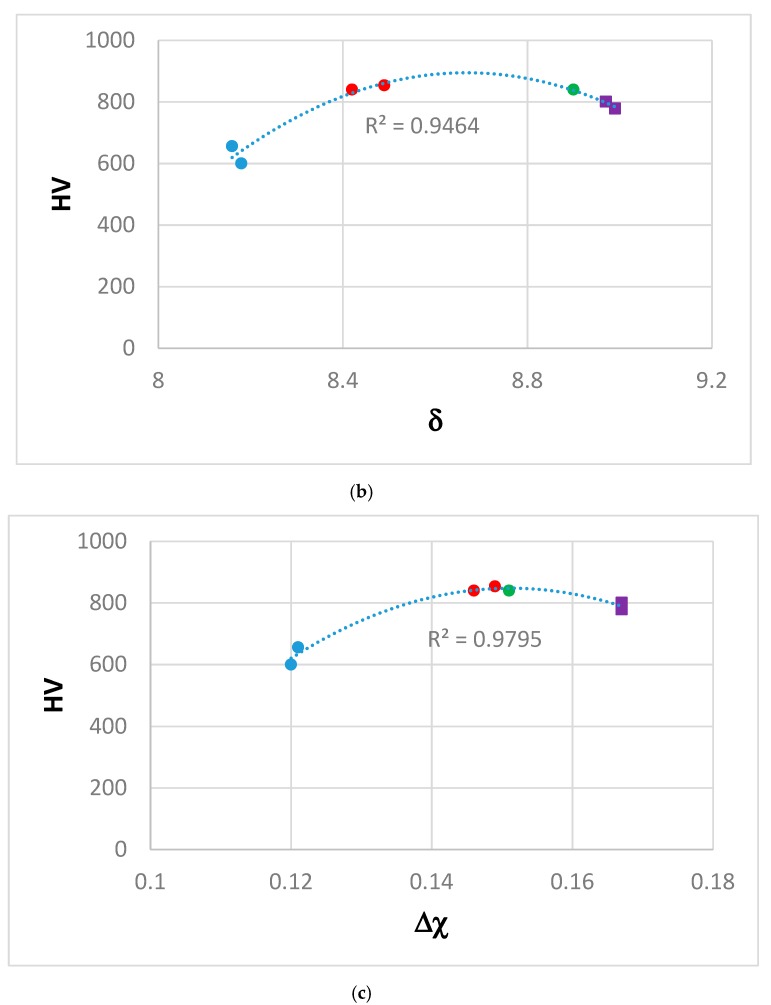
Vickers hardness (HV) versus VEC (**a**) or δ (**b**) or Δχ (**c**) of the alloys KZ5 = Nb-24Ti-18Si-5Al-5Cr [12] (blue), JN1 = Nb-24Ti-18Si-5Al-5Cr-5Hf [46] (green), ZF6 (red) and ZF9 (purple).

The alloying behaviour of Nb-silicide based alloys and of the phases that can be present in their microstructures, and properties of the alloys and their phases can be studied using the parameters VEC, δ and Δχ [3,4,8,35,36]. In Figure 8 we show how the hardness of the alloys KZ5 (Nb-24Ti-18Si-5Al-5Cr), JN1 (Nb-24Ti-18Si-5Al-5Cr-5Hf [46]), ZF6 and ZF9 varies with the alloy parameters VEC, δ and Δχ. Data for the alloys KZ5 and JN1 has been included in order to show how the additions of Hf and/or Ge to the “basis” alloy KZ5 affect hardness. The Nb_5_Si_3_ was facetted in the alloys KZ5, JN1 and ZF6. The Hf-rich Nb_5_Si_3_ in ZF9 was also faceted (Figure 5b). The hardness increased as the alloy VEC value decreased and the values of the alloy parameters δ and Δχ increased. The alloy KZ5 had 48–55 vol.% Nb_ss_ and 45-52 vol.% Nb_5_Si_3_ [12], the alloy JN1 had 42 vol.% Nb_ss_ and 58 vol.% Nb_5_Si_3_ [46] and the alloy ZF6-HT had 17.3 vol.% Nb_ss_ and 82.7 vol.% Nb_5_Si_3_. Constraint effects on hardness owing to the faceting of Nb_5_Si_3_ apply to all alloys. The hardness increased as the vol.% of Nb_5_Si_3_ increased compared with the alloy KZ5. All the alloys fall on the same curve with high R^2^ values in all cases in the HV versus VEC, δ and Δχ plots in Figure 8. Remarkably, the alloy ZF9 falls in the same curve with the alloys KZ5, JN1 and ZF6 even though only its Hf-rich Nb_5_Si_3_ was facetted. The alloy ZF9 also had a higher vol.% of Nb_5_Si_3_.

## 5. Conclusions 

In this work we studied the effects of the synergy of Ti, Ge, Cr, Al and Hf additions on the microstructure and hardness of the as cast and heat-treated alloys Nb-24Ti-18Si-5Al-5Cr-Ge (ZF6) and Nb-24Ti-18Si-5Al-5Cr-5Ge-5Hf (ZF9). We also compared these alloys with RM bcc solid solution + intermetallic HEAs. The partitioning of Al, Cr, Ge, Hf and Ti between the phases that were present in the microstructures contributed to the macrosegregation of Si, Ti, Cr and Al in both alloys. Alloying with Hf reduced but did not eliminate the macrosegregation of Si. Chromium was the key element in the macrosegregation of Ti. The microstructures of the cast alloys consisted of the bcc Nb_ss_, Nb_5_Si_3_ silicide and C14-NbCr2 Laves phase and the βNb_5_Si_3_ was the primary phase. The Nb_ss_ + βNb_5_Si_3_ eutectic was formed only in ZF6. The microstructures of the heat-treated alloys consisted of the Nb_ss_, Nb_5_Si_3_ phases. The transformation of βNb_5_Si_3_ to αNb_5_Si_3_ was not completed after 100 h at 1400 °C. Contamination of the alloys by oxygen during heat treatment led to the formation of Ti and Hf oxides respectively in ZF6 and ZF9. Alloying with Hf decreased the hardness of the alloy ZF9. The hardness of the Nb_5_Si_3_ silicide decreased to that of the unalloyed silicide after 100 h at 1400 °C in the alloy ZF9.

## Figures and Tables

**Table 1:** Density and % areas of Nb_ss_ in as cast (AC) and heat treated (HT) alloys Nb-24Ti-18Si-5Ge-5Cr-5Al (ZF6) and Nb-24Ti-18Si-5Ge-5Cr-5Al-5Hf (ZF9).

Alloy	Density (g/cm^3^)	% Area		
		Top	Bulk	Bottom
ZF6-AC	6.54 ± 0.01	22.4 ± 1.8	17.1 ± 3.7	29.2 ± 2.8
ZF6-HT	-	-	17.3 ± 3.5	-
ZF9-AC	6.96 ± 0.02	-	-	-
ZF9-HT	-	-	-	-

**Table 2:** Chemical analysis data (at.%) of as cast (AC) and heat treated (HT) alloy ZF6.

Area/Phase	Nb	Ti	Si	Ge	Cr	Al
ZF6-AC	-	-	-	-	-	-
Top*	41.6 ± 0.4	26.2 ± 0.1	16.1 ± 0.1	4.9 ± 0.1	5.4 ± 0.2	5.8 ± 0.6
	41.4–42.0	26.1–26.2	16.0–16.1	4.8–5.0	5.2–5.5	5.3–6.2
Bulk*	41.9 ± 1.0	24.9 ± 0.8	18.0 ± 1.9	5.1 ± 0.3	4.5 ± 1.0	5.6 ± 1.1
	40.9–43.3	23.9–26.0	16.1–20.1	4.9–5.6	3.5–5.6	4.1–6.6
Bottom*	41.1 ± 0.4	26.1 ± 0.4	16.3 ± 0.4	5.4 ± 0.1	5.2 ± 0.2	6.1 ± 0.5
	40.7–41.6	25.5–26.3	15.8–16.6	5.3–5.5	5.0–5.4	5.9–6.7
Nb_ss_	52.3 ± 1.6	27.9 ± 0.7	2.1 ± 0.2	1.7 ± 0.3	9.2 ± 1.1	6.8 ± 0.4
	50.2–53.6	27.0–28.8	2.0–2.4	1.4–2.2	8.0–10.6	6.6–7.3
Ti-rich Nb_ss_	45.4 ± 3.1	31.3 ± 1.4	1.8 ± 0.4	1.6 ± 0.3	12.9 ± 1.2	7.0 ± 0.3
	42.0–48.7	29.9–32.7	1.3–2.1	1.3–1.9	11.5–14.4	6.7–7.7
βNb_5_Si_3_	42.4 ± 1.1	22.1 ± 0.9	23.2 ± 1.3	7.3 ± 0.4	1.4 ± 0.2	3.6 ± 1.0
	41.3–43.9	20.9–23.0	22.1–24.8	6.9–7.9	1.1–1.5	2.3–4.8
Ti-rich βNb_5_Si_3_	34.1 ± 0.4	29.5 ± 0.3	20.9 ± 1.4	8.4 ± 0.4	2.4 ± 0.4	4.7 ± 0.7
	33.8–34.5	29.1–29.7	19.3–27.1	8.1–8.9	2.1–2.8	4.1–5.4
C14-Cr_2_Nb^+^	18.4	24.8	6.2	1.2	46.3	3.1
Eutectic	45.5 ± 1.2	28.4 ± 0.6	8.1 ± 1.5	3.7 ± 0.5	8.0 ± 1.1	6.3 ± 0.4
	44.2–46.9	27.7–29.0	5.8–9.0	3.2–4.3	6.6–9.3	6.0–6.8
ZF6-HT* (1400 °C/100 h)	43.1 ± 1.0	24.3 ± 1.2	17.4 ± 0.8	5.8 ± 0.2	4.2 ± 0.3	5.2 ± 0.8
	41.9–44.6	23.0–25.9	16.4–18.7	5.5–6.2	4.0–4.7	4.1–6.1
Nb_ss_	52.6 ± 0.5	26.9 ± 0.3	0.9 ± 0.5	1.0 ± 0.2	12.3 ± 0.2	6.3 ± 0.4
	52.1–53.0	26.4–27.2	0–1.2	0.8–1.2	11.9–12.4	5.7–6.7
Nb_5_Si_3_	42.2 ± 0.9	21.7 ± 0.9	23.1 ± 1.2	7.4 ± 0.5	1.8 ± 0.4	3.8 ± 0.6
	41.4–43.4	20.5–22.6	21.7–24.4	7.0–8.1	1.5–2.3	3.1–4.3
Ti-rich Nb_5_Si_3_	37.6 ± 1.1	26.1 ± 1.1	21.0 ± 0.3	7.8 ± 0.2	2.7 ± 0.1	4.8 ± 0.3
	35.9–39.1	25.0–27.9	20.8–21.6	7.6–8.0	2.5–2.8	4.6–5.2

^+^ This was the average of three analyses; * large area analysis.

**Table 3:** Chemical analysis data (at.%) of as cast (AC) and heat treated (HT) alloy ZF9.

Area/Phase	Nb	Ti	Si	Ge	Cr	Al	Hf
ZF9-AC	-	-	-	-	-	-	-
Top*	35.6 ± 0.1	27.3 ± 0.2	16.0 ± 0.6	5.3 ± 0.2	5.9 ± 0.3	4.4 ± 0.3	5.5 ± 0.1
	35.7–35.9	27.0–27.5	15.3–16.5	5.0–5.4	5.5–6.1	4.1–4.7	5.5–5.6
Bulk*	36.7 ± 0.4	24.8 ± 0.6	17.6 ± 0.8	5.1 ± 0.2	4.6 ± 0.2	5.8 ± 0.4	5.4 ± 0.2
	36.3–37.3	24.1–25.5	16.4–18.4	4.9–5.4	4.3–4.9	5.2–6.1	5.2–5.6
Bottom*	35.3 ± 0.1	27.1 ± 0.2	15.5 ± 0.1	5.5 ± 0.5	6.2 ± 0.3	5.0 ± 0.2	5.4 ± 0.2
	35.2–35.4	27.0–27.3	15.4–15.6	5.1–6.0	5.9–6.4	4.9–5.2	5.2–5.5
Nb_ss_	40.4 ± 3.4	31.5 ± 2.3	1.6 ± 0.2	1.0 ± 0.2	15.0 ± 1.2	7.8 ± 0.3	2.7 ± 0.2
	35.5–44.4	29.1–35.1	1.5–1.8	0.7–1.1	13.4–16.1	7.5–8.4	2.5–3.0
βNb_5_Si_3_	37.2 ± 0.2	21.0 ± 0.3	24.2 ± 0.5	6.6 ± 0.1	1.2 ± 0.1	5.0 ± 0.4	4.8 ± 0.1
	36.8–37.3	20.6–21.2	23.8–25.1	6.5–6.8	1.2–1.3	4.4–5.5	4.7–4.9
Hf-rich Nb_5_Si_3_	30.9 ± 0.7	24.0 ± 0.3	24.5 ± 0.6	7.0 ± 0.3	0.8 ± 0.2	3.8 ± 0.6	9.0 ± 0.2
	29.9–31.8	23.6–24.2	23.7–25.1	6.6–7.4	0.5–0.9	3.3–4.8	8.8–9.2
Cr_2_Nb^+^	20.5	19.7	5.6	1.3	43.7	4	5.2
ZF9-HT*	38.0 ± 2.0	24.2 ± 1.5	17.1 ± 1.3	5.2 ± 0.2	4.8 ± 0.6	5.5 ± 0.8	5.2 ± 0.2
	36.4–40.2	22.8–25.7	15.8–18.4	5.0–5.4	4.1–5.3	4.9–6.4	5.0–5.4
Nb_ss_	56.6 ± 3.3	25.3 ± 1.2	0.7 ± 0.5	0.6 ± 0.6	11.8 ± 0.8	3.8 ± 1.2	1.2 ± 0.1
	56.8–59.8	24.3–26.7	0.0–1.4	0.0–1.2	10.6–12.6	2.4–5.4	1.1–1.3
Nb_5_Si_3_	37.0 ± 0.6	21.2 ± 0.4	24.8 ± 0.5	6.7 ± 0.3	2.3 ± 0.3	3.2 ± 0.4	4.8 ± 0.2
	36.4–37.9	20.6–21.7	24.2–25.2	6.3–7.2	2.0–2.7	2.7–3.4	4.5–4.9
Hf-rich Nb_5_Si_3_	30.2 ± 1.2	24.3 ± 0.9	25.4 ± 0.6	7.0 ± 0.2	1.0 ± 0.3	3.3 ± 0.5	8.8 ± 0.1
	29.0–31.3	23.6–25.3	25.0–26.1	6.8–7.2	0.7–1.3	2.7–3.6	8.8–8.9

^+^ This was the average data of three measurements, * large area analysis.

**Table 4:** Lattice parameter (Å) of the Nb_ss_ in the alloys ZF6 and ZF9.

Alloy	ZF6-AC	ZF6-HT	ZF9-AC	ZF9-HT
Parameter	3.252	3.260	3.256	3.264

**Table 5:** Vickers hardness (HV) of the as cast and heat-treated alloys ZF6 and ZF9.

Alloy	Hardness	Microhardness	
		Nb_ss_	Nb_5_Si_3_
ZF6-AC	840 ± 34	—^+^	1644 ± 89
ZF6-HT	854 ± 31	691 ± 37	1576 ± 60
ZF9-AC	801 ± 37	—^+^	1495 ± 88
ZF9-HT	779 ± 11	—^+^	1391 ± 21

^+^ Not measured owing to the small size of the Nb_ss_ in the alloy.

**Table 6:** Synergistic effect of alloying elements on macrosegregation of Si in as cast Nb-18Si silicide-based alloys.

Elements in Synergy	Alloy Code* & Reference	C_max_^Si^–C_min_^Si^	Macrosegregation
Ti + Cr + Ge	ZF4 [15]	5.3	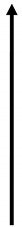
Ti + Cr + Al + Ge	ZF6	4.3	
Ti + Ge	ZF3 [18]	3.2	
Ti + Cr + Al + Ge + Hf	ZF9	3.1	
Ti + Al + Ge	ZF5 [13]	2.9	
Al + Ge	ZF8 [13]	2.5	
Ti + Al	KZ7 [12]	2.3	
Ge	ZF1 [9]	2.0	
Ti + Cr	KZ4 [12]	1.9	
Ti	KZ3 [12]	1.6	
Cr + Ge	ZF7 [15]	1.5	
Ti + Cr + Al	KZ5 [12]	1.3	

* see Appendix A.

**Table 7:** Effect of alloying on macrosegregation of Al, Cr and Ti in as cast Nb-18Si silicide-based alloys.

Alloying Additions	Alloy Code* & Reference	C_max_^i^–C_min_^i^ (i = Ti, Cr, Al)		
		Ti	Cr	Al
Ti + Ge + Cr + Al + Hf	ZF9	3.4	2.1	2
Ti + Ge + Cr	ZF4 [15]	3.3	2.2	-
Ti + Ge + Cr + Al	ZF6	2.4	2.1	2.6
Ti + Al	KZ7 [12]	2.3	-	-
Ti + Cr + Al	KZ5 [12]	1.4	-	-
Ti + Ge	ZF3 [18]	1.4	-	-
Ti + Cr	KZ4 [12]	1.4	1.8	-
Ti	KZ3 [12]	3.1	-	-

* see Appendix A.

**Table 8:** Concentration of Si + Ge + Al in the Nb_ss_ + βNb_5_Si_3_ eutectic in the as cast alloys ZF5, ZF6 and ZF8.

**Alloy Code**	**Nominal Composition (at.%)**	**Si + Ge +Al in The Eutectic (at.%)**	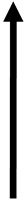
ZF8-AC [13]	Nb-18Si-5Ge-5Al	22.6	
ZF5-AC [13]	Nb-24Ti-18Si-5Ge-5Al	18.8	
ZF6-AC	Nb-24Ti-18Si-5Ge-5Cr-5Al	18.1	

**Table 9:** Concentration of Si + Ge in the Nb_ss_ + βNb_5_Si_3_ eutectic in as cast ZF series alloys.

**Alloy Code & Reference**	**Nominal Composition (at.%)**	**Si + Ge in The Eutectic (at.%)**	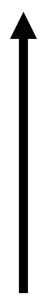
ZF1-AC [9]	Nb-18Si-5Ge	17.7	
ZF2-AC [9]	Nb-18Si-10Ge	17.6	
ZF8-AC [13]	Nb-18Si-5Ge-5Al	16.3	
ZF7-AC [15]	Nb-18Si-5Ge-5Cr	15.2	
ZF4-AC [15]	Nb-24Ti-18Si-5Ge-5Cr	14.8	
ZF5-AC [13]	Nb-24Ti-18Si-5Ge-5Al	12.6	
ZF6-AC	Nb-24Ti-18Si-5Ge-5Cr-5Al	11.8	

**Table 10:** The parameters ΔH^chem^, ΔS_mix_, VEC, δ, Δχ and Ω of the alloys ZF6 and ZF9 and their solid solutions in the as cast (AC) and heat treated (HT) conditions. For calculation of parameters see [35,36]. Note that the symbol Q was used instead of Ω in [36].

Condition	Alloy	Nb_ss_	Parameter					
-	-	-	ΔH^chem^ (Kj·mol^−1^)	ΔS_mix_ (J·mol^−1^·K)	VEC	δ	Δχ	Ω
AC	ZF6	Nb_ss_ Ti-rich Nb_ss_	−39.4	12.31	4.458	8.42	0.146	0.672
			−12.87	10.38	4.639	5.12	0.078	1.855
			−13	10.9	4.642	5.52	0.077	1.888
HT	ZF6	Nb_ss_	−40.4	12.16	4.463	8.49	0.149	0.650
			−9.7	10.07	4.709	5.02	0.063	2.411
AC	ZF9	Nb_ss_	−40.09	13.65	4.419	8.97	0.167	0.730
			−12.29	11.83	4.626	5.95	0.084	2.146
HT	ZF9	Nb_ss_	−40.73	13.52	4.421	8.99	0.167	0.714
			−6.87	9.68	4.764	4.96	0.063	3.367

## Appendix A

**Table A1:** Nominal compositions (at.%) of alloys that are referred to in this paper

Alloy Code	Nominal Composition (at.%)	Reference
KZ3	Nb-24Ti-18Si	[12]
KZ4	Nb-24Ti-18Si-5Cr	[12]
KZ7	Nb-24Ti-18Si-5Al	[12]
KZ5	Nb-24Ti-18Si-5Al-5Cr	[12]
ZF1	Nb-18Si-5Ge	[9]
ZF2	Nb-18Si-10Ge	[9]
ZF3	Nb-24Ti-18Si-5Ge	[18]
ZF7	Nb-18Si-5Cr-5Ge	[15]
ZF4	Nb-24Ti-18Si-5Cr-5Ge	[15]
ZF8	Nb-18Si-5Al-5Ge	[13]
ZF5	Nb-24Ti-18Si-5Al-5Ge	[13]
